# Supplementary material for: Cationic nanoparticle as an inhibitor of cell-free DNA-induced inflammation
Source: Nat Commun. 2018 Oct 16;9:4291. doi: 10.1038/s41467-018-06603-5 (PMC6191420; doi:10.1038/s41467-018-06603-5)
Supplement: Supplementary file 1 — Supplementary Information [file 41467_2018_6603_MOESM1_ESM.pdf]

SUPPLEMENTARY INFORMATION

Cationic Nanoparticles as DNA Induced Inflammatory Inhibitors to Treat Rheumatoid Arthritis

Huiyi Liang[1], Bo Peng[1], Cong Dong[1], Lixin Liu[1]*, Jiaji Mao[2], Song Wei[3], Xinlu Wang[3], Hanshi Xu[4], Jun Shen[2]*, Hai-Quan Mao[1,5], Xiaohu Gao[1,6], Kam W. Leong[1,7]*, Yongming Chen[1]*

Table of Contents of Supplementary Information

Supplementary Figure 1. Synthesis of PDMA and PLGA-*b*-PDMA and particle morphology of cNP. 2

Supplementary Figure 2. cNP has a lower cytotoxicity than PDMA and the cationic materials alone show no significant stimulus to cells. 3

Supplementary Figure 3. Cationic materials could inhibit nucleic acid-mediated activation of TLRs. 4

Supplementary Figure 4. MyD88 and TRAF6 enhanced expression with CpG were down-regulated by cationic materials in Ramos Blue^{TM} cells. 5

Supplementary Figure 5. Cationic materials can reduce the cellular uptake of immuno-stimulatory nucleic acid. 6

Supplementary Figure 6. Cationic materials interacted with intracellular nucleic acid. 7

Supplementary Figure 7. Morphology and iconography study of mice treatment by cationic materials. 8

Supplementary Figure 8. Morphology and iconography study in both early and established therapeutic treatment of rats. 9

Supplementary Figure 9. Biodistribution of cationic materials in normal rats and CIA rats (score 2) via i.v. injection. 11

Supplementary Figure 10. Quantification of TNF-a, IL-6 and MMP-3 immunoreactive cells in synovial tissue of normal, model, PDMA, and cNP groups. 12

Supplementary Figure 11. In vivo acute toxicity study of cationic polymers in rats. 13

Supplementary Figure 12. In vivo long-term toxicity of cationic materials in rats. 14

Supplementary Table 1. Basic information and clinical characteristics of RA patients. 15

Supplementary References 15

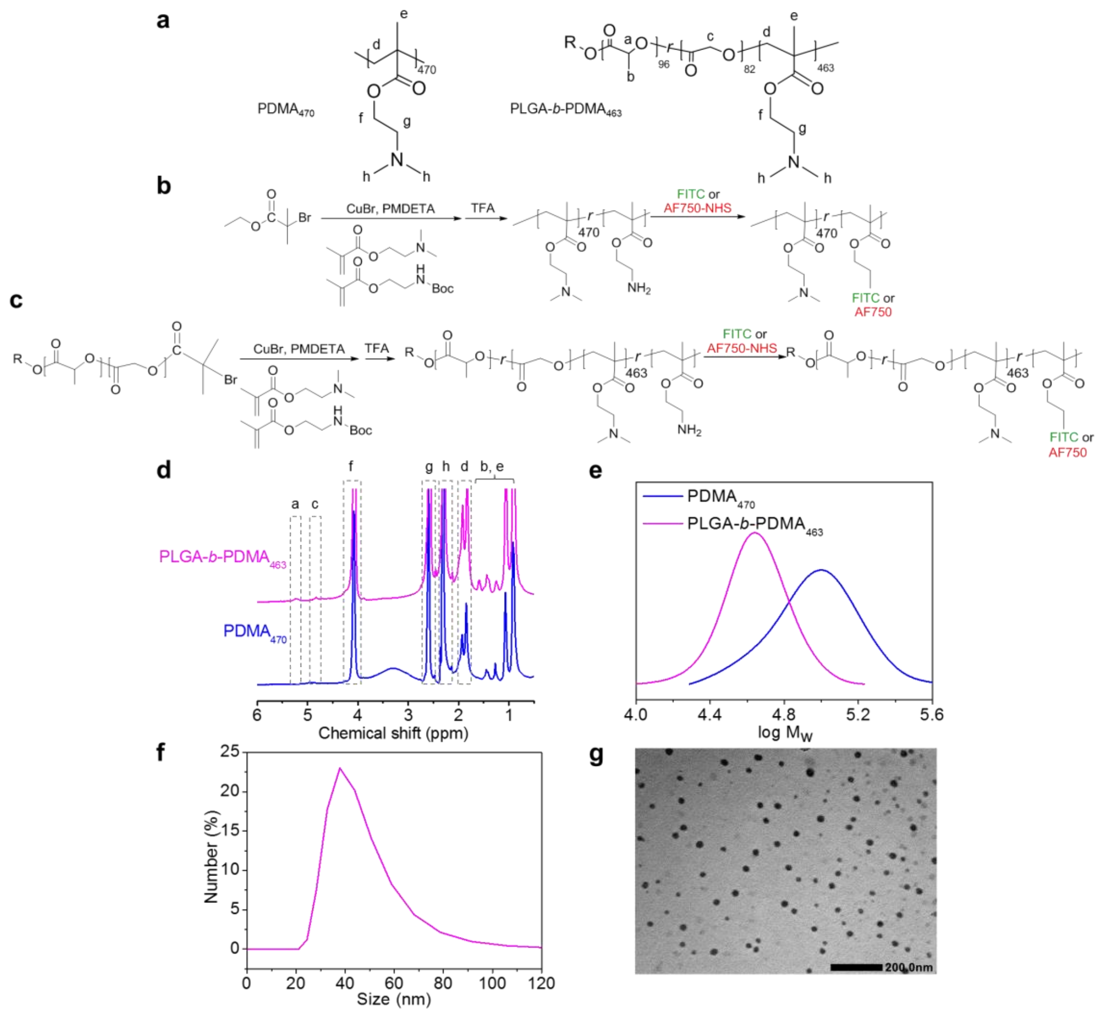

Supplementary Figure 1. Synthesis of PDMA and PLGA-*b*-PDMA and particle morphology of cNP. a, Chemical structure of $PDMA_{470}$ and PLGA-*b*-$PDMA_{463}$ block copolymer. **b,** Schematic syntheses of $PDMA_{470}$ homopolymer and dye-conjugated $PDMA_{470}$ homopolymer. **c,** Schematic syntheses of PLGA-*b*-$PDMA_{463}$ block copolymer and dye-conjugated PLGA-*b*-$PDMA_{463}$ block copolymer. **d,** ^{1}H NMR spectrum of $PDMA_{470}$ and PLGA-*b*-$PDMA_{463}$ after purification in $CDCl_3$. **e,** GPC traces of $PDMA_{470}$ and PLGA-*b*-$PDMA_{463}$ in DMF eluent, using PS standard for analysis. **f,** Number average distribution curve of cNP in PBS measured by DLS. **g,** TEM micrograph of cNP.

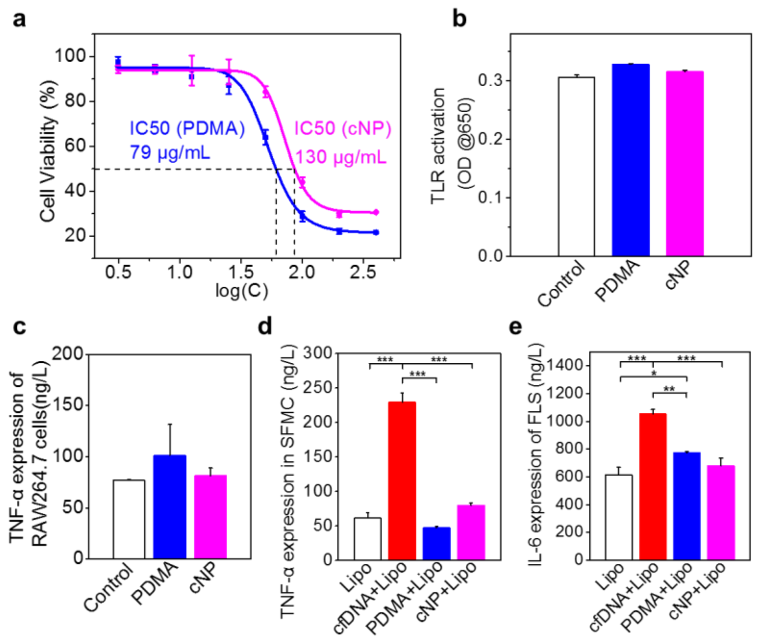

Supplementary Figure 2. cNP has a lower cytotoxicity than PDMA and the cationic materials alone show no significant stimulus to cells. a, Cell viability of RAW264.7 cells treated with different cationic materials at various concentrations for 24 h incubation. **b,** TLR activation of Ramos Blue^{TM} cells after adding 50 μg/mL cationic materials to the medium for 24 h shows that cationic materials could not activate TLR. **c,** 25 μg/mL cationic materials could not raise TNF-α expression in RAW264.7 cells. **d,** Cationic materials could not raise TNF-α expression in patient's SFMC. After incubation with 25 μg/mL PDMA or cNP for 24 h, TNF-α expression had no significant increase relative to the control group incubated with Lipofectamine® 2000 (Lipo) only. **e,** 0.5 μg/mL cationic materials could not raise IL-6 expression in patient's FLS after 24 h incubation. In **b**, **c**, **d** and **e**, statistical significance was calculated by one-way ANOVA with the LSD post-test, * $0.01<P<0.05$, ** $0.001<P<0.01$, *** $P<0.001$. In **a-e**, data are presented as the mean ± s.e.m.

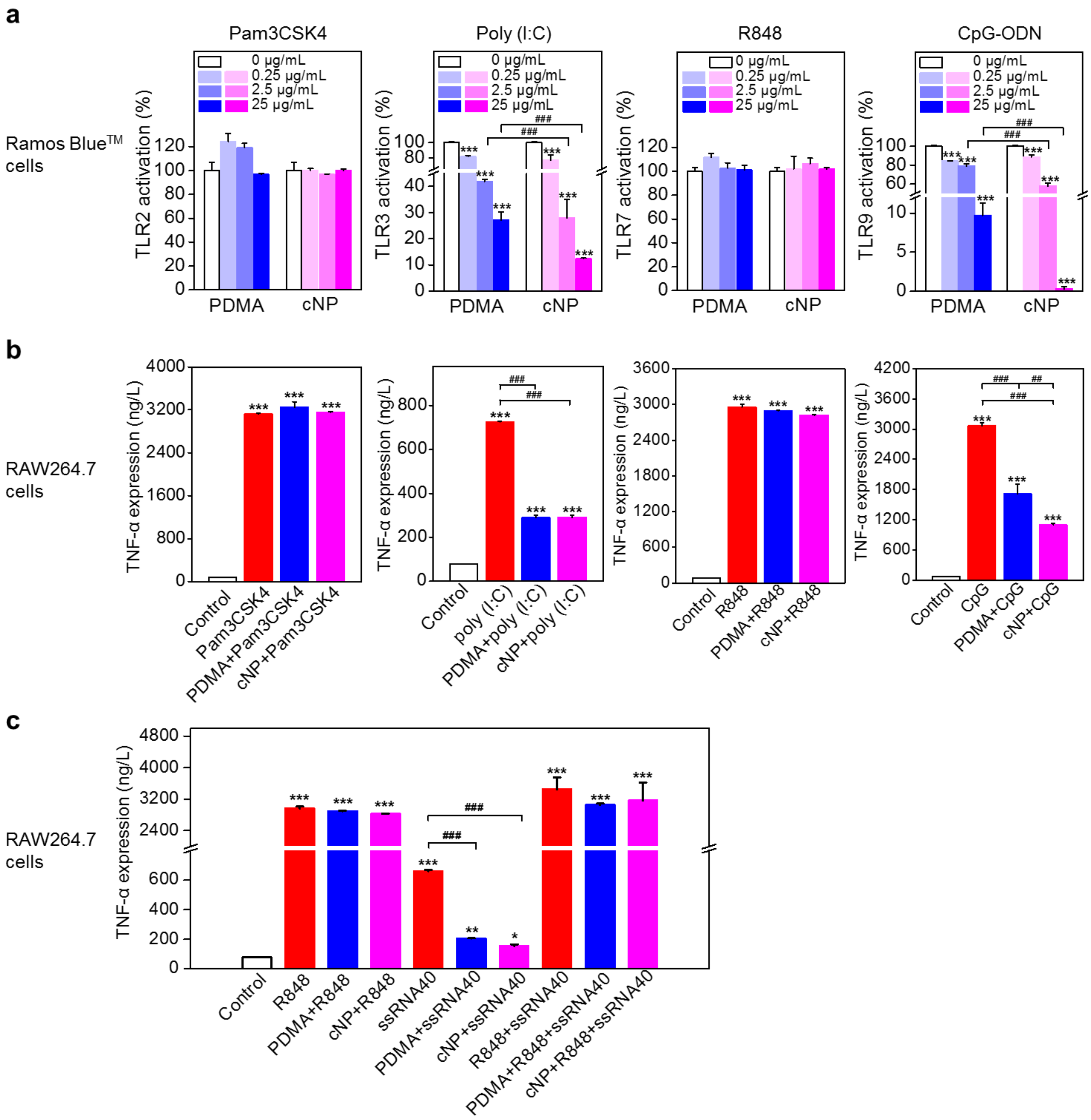

Supplementary Figure 3. Cationic materials could inhibit nucleic acid-mediated activation of TLRs. a and **b.** Cationic materials inhibited the TLR activation stimulated by synthetic nucleic acid-based TLR agonists such as poly (I:C) (TLR3) and CpG (TLR9), but did not inhibit the activation stimulated by the synthetic non-nucleic acid TLR agonists such as Pam3CSK4 (TLR2) and R848 (TLR7) in (**a**) Ramos Blue™ cells, and (**b**) RAW264.7 cells. **c.** Cationic materials inhibited TLR7 activation stimulated by nucleic acid-based agonist (ssRNA40) but not the non-nucleic acid-based agonist (R848) in RAW264.7 cells. In **a-c,** Statistical significance was calculated by one-way ANOVA with the LSD post-test, * 0.01<*P*<0.05, ** 0.001<*P*<0.01, *** *P*<0.001 versus 0 µg/mL materials+ CpG (**a**) or control (**b**, **c**). ## 0.001<*P*<0.01, ###*P* < 0.001 between two groups. Data are presented as the mean ± s.e.m.

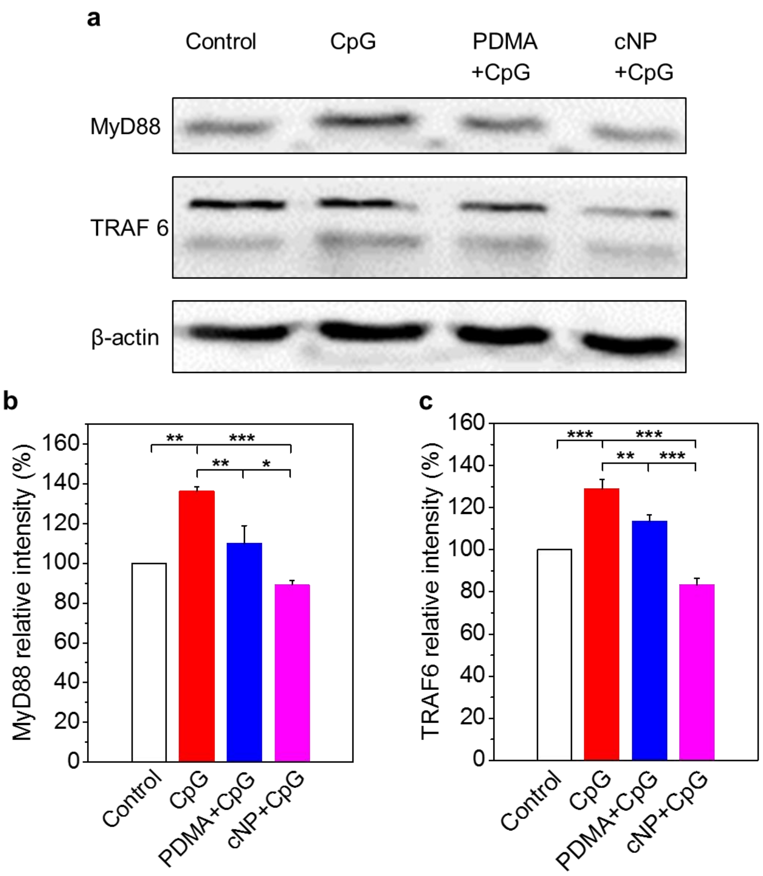

Supplementary Figure 4. MyD88 and TRAF6 enhanced expression with CpG were down-regulated by cationic materials in Ramos Blue™ cells. a, Ramos Blue™ cells were stimulated with CpG 2006 with or without cationic materials for 24 h, then cells were fractionated and extracts were western-blotted. Both MyD88 and TRAF6 proteins had an up-regulation in CpG group but had down-regulation in cationic materials treated groups. **b** and **c,** Semi-quantitative analysis of western-blot in Supplementary Figure 2a. β-actin was used as a loading control and immunoblot band intensities were quantified using loading controls. Statistical significance was calculated by one-way ANOVA with the LSD post-test, * $0.01<P<0.05$, ** $0.001<P<0.01$, *** $P<0.001$. Data are presented as the mean ± s.e.m.

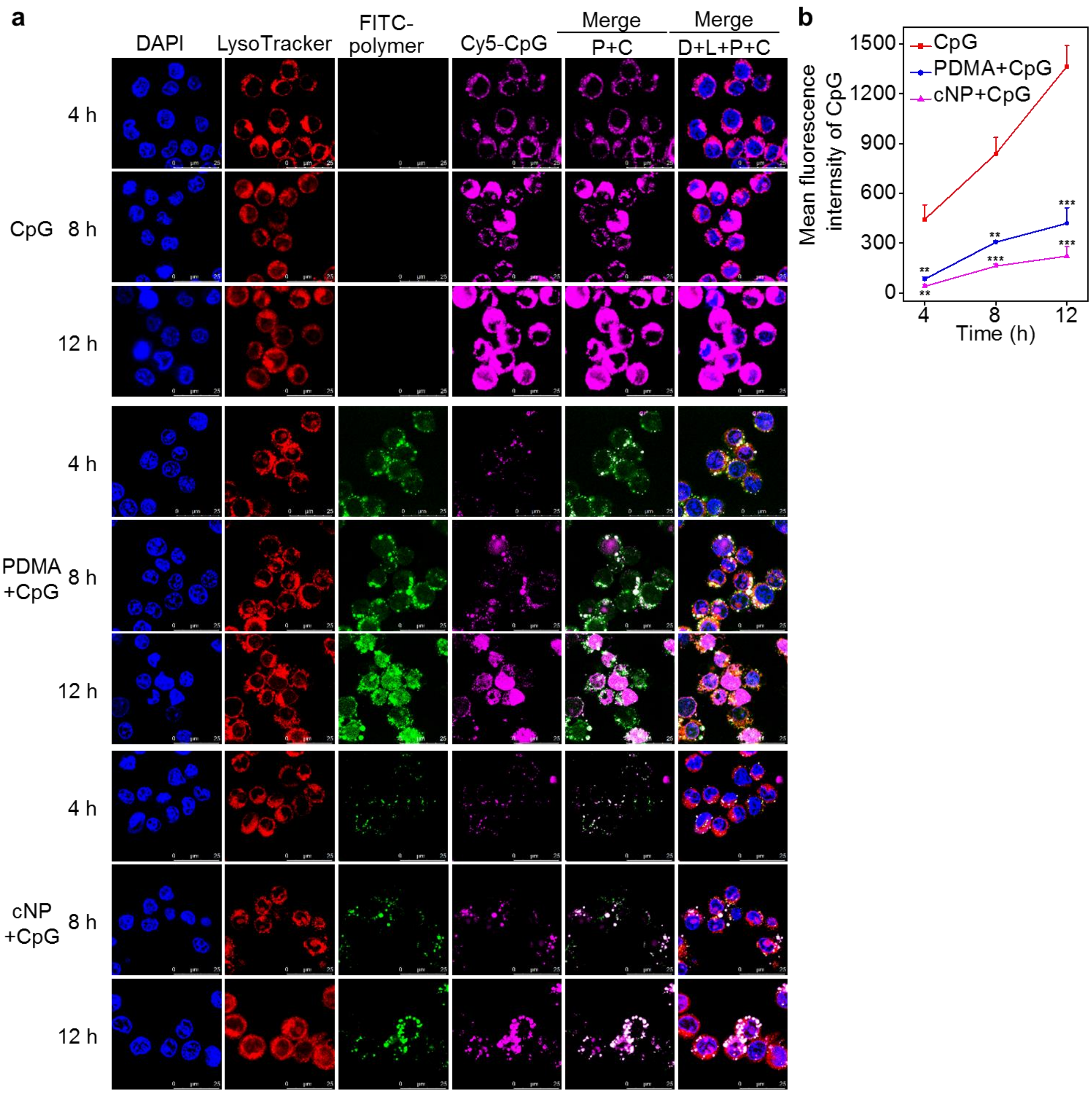

Supplementary Figure 5. Cationic materials can reduce the cellular uptake of immuno-stimulatory nucleic acid. a, Evaluation of intracellular trafficking of cationic materials and the immuno-stimulatory nucleic acids at different time points. CpG 1826 and polymers were added to RAW264.7 cells at the same time then incubated at 37 ℃ for 12 h. The intracellular localization of CpG and cationic materials after incubation for 4 h, 8h and 12h was observed by confocal microscopy (×1800, scale bar: 25 μm). Colocalization of CpG and cationic materials showed up as white spots in the merged images. D, L, P, and C indicates DAPI, LysoTracker, polymer, and CpG, respectively. **b,** Quantification of mean fluorescence intensity of Cy5-CpG 1826 in RAW cells after incubation with cationic materials, calculated by Image J software. Statistical significance was calculated by one-way ANOVA with the LSD post-test, ** 0.001<P<0.01, *** P<0.001 versus CpG group. Data are presented as the mean ± s.e.m.

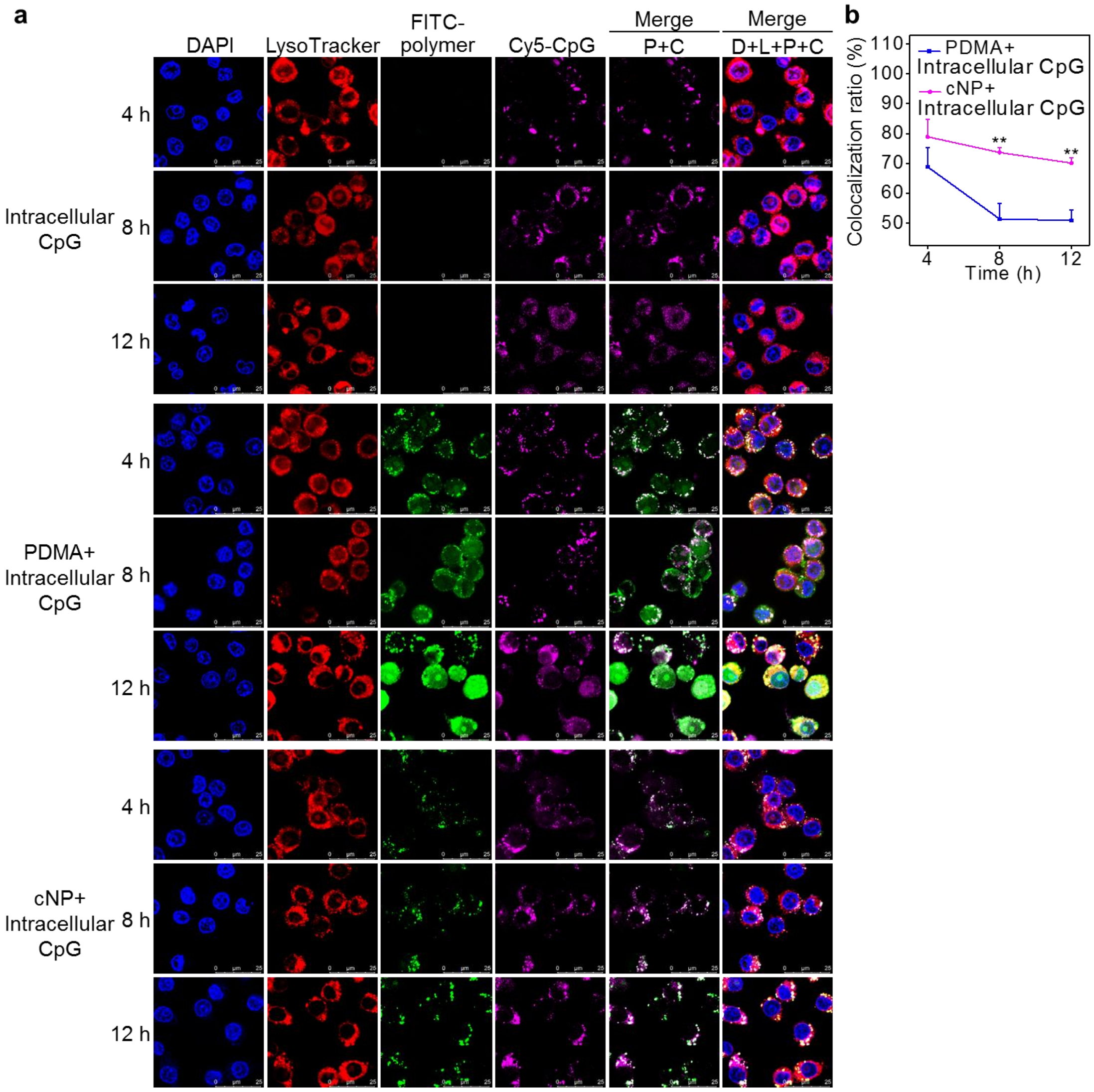

Supplementary Figure 6. Cationic materials interacted with intracellular nucleic acid. a, Evaluation of colocalization of cationic materials with the internalized nucleic acids at different time points. Firstly, RAW264.7 cells were incubated at 37 ℃ for 4 h with Cy5-CpG 1826. After removal of the supernatant and extensive washing, fresh culture medium and cationic materials were added. The intracellular localization of CpG and cationic materials after incubation for 4 h, 8h and 12h was observed by confocal microscopy (×1800, scale bar: 25 μm). Colocalization of intracellular CpG and cationic materials showed up as white spots in the merged images. D indicates DAPI, L indicates LysoTracker, P indicates polymer, and C indicates CpG. **b,** Quantitative colocalization ratio of Cy5-CpG 1826 and different FITC-cationic materials at various time course, calculated by Leica XPS software. The colocalization ratio was the ratio of colocalization area (the area of white spots) and foreground area (the area of green and purple spots). Statistical significance was calculated by one-way ANOVA with the LSD post-test, ** $0.001<P<0.01$ versus PDMA+Intracelluar CpG group. Data are presented as the mean ± s.e.m.

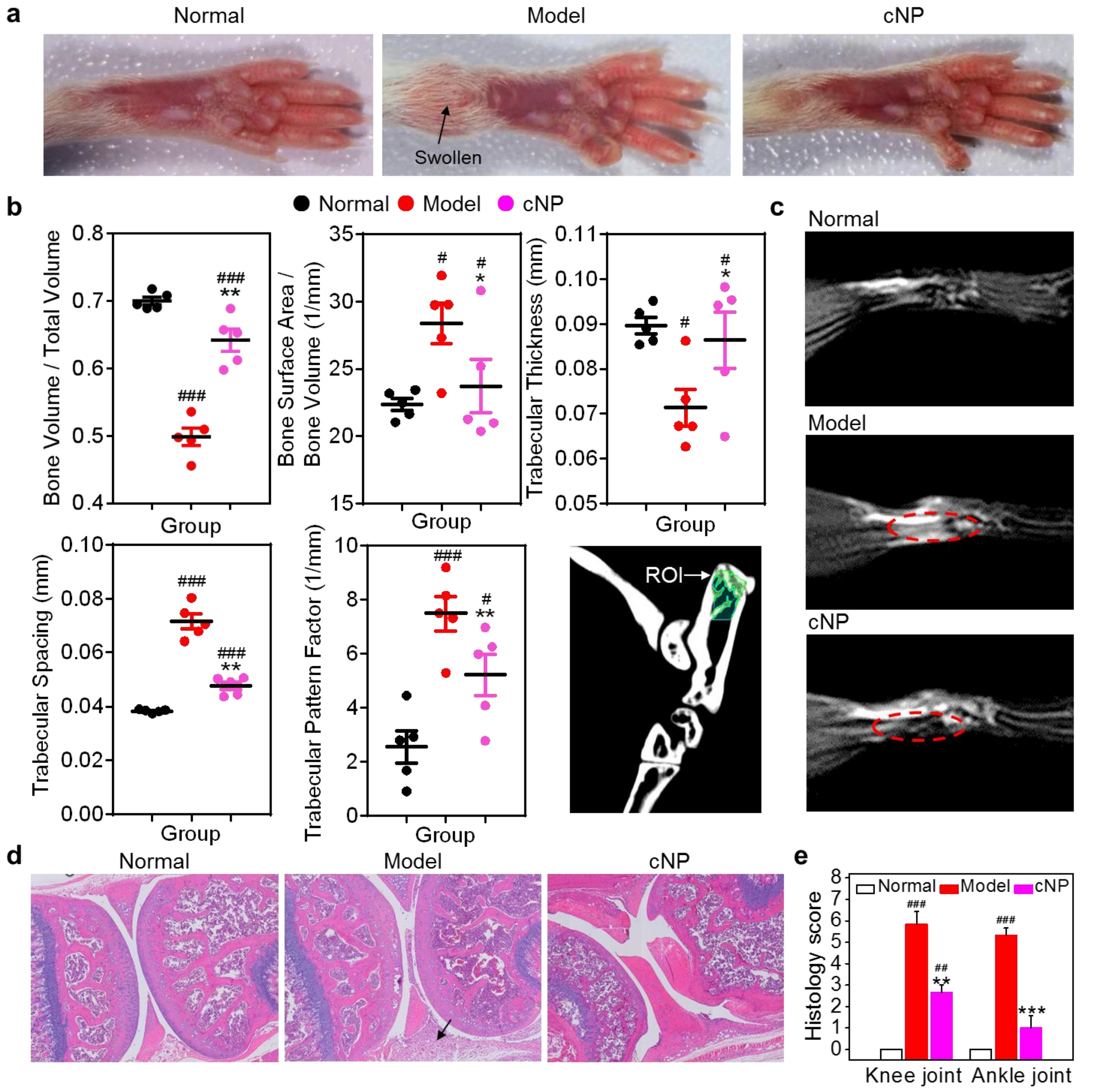

Supplementary Figure 7. Morphology and iconography study of mice treatment by cationic materials. a, Rear views of joint swelling of mice at day 5. **b,** Analysis of ankle joint bone histomorphometric parameters using micro CT data. The bone trabecular of calcaneus was chosen as region of interest (ROI). **c,** Representative T2-weighted MRI images of the ankle joints of mice at day 7. Red circles indicate effusion in ankle joint. **d,** Representative H&E staining of the knee joints of mice at day 8. Some inflammatory cell infiltration in the synovium (single arrow) was visible in the RA model group but not the other two groups (×200). **e,** Average histology scores of knee joints and ankle joints of mice in different groups. In **b** and **e,** statistical significance was calculated by one-way ANOVA with the LSD post-test, * 0.01<*P*<0.05, ** 0.001<*P*<0.01, *** *P*<0.001 versus the model group; # 0.01<*P*<0.05, ### *P*<0.001 versus the normal group. Data are presented as the mean ±s.e.m.

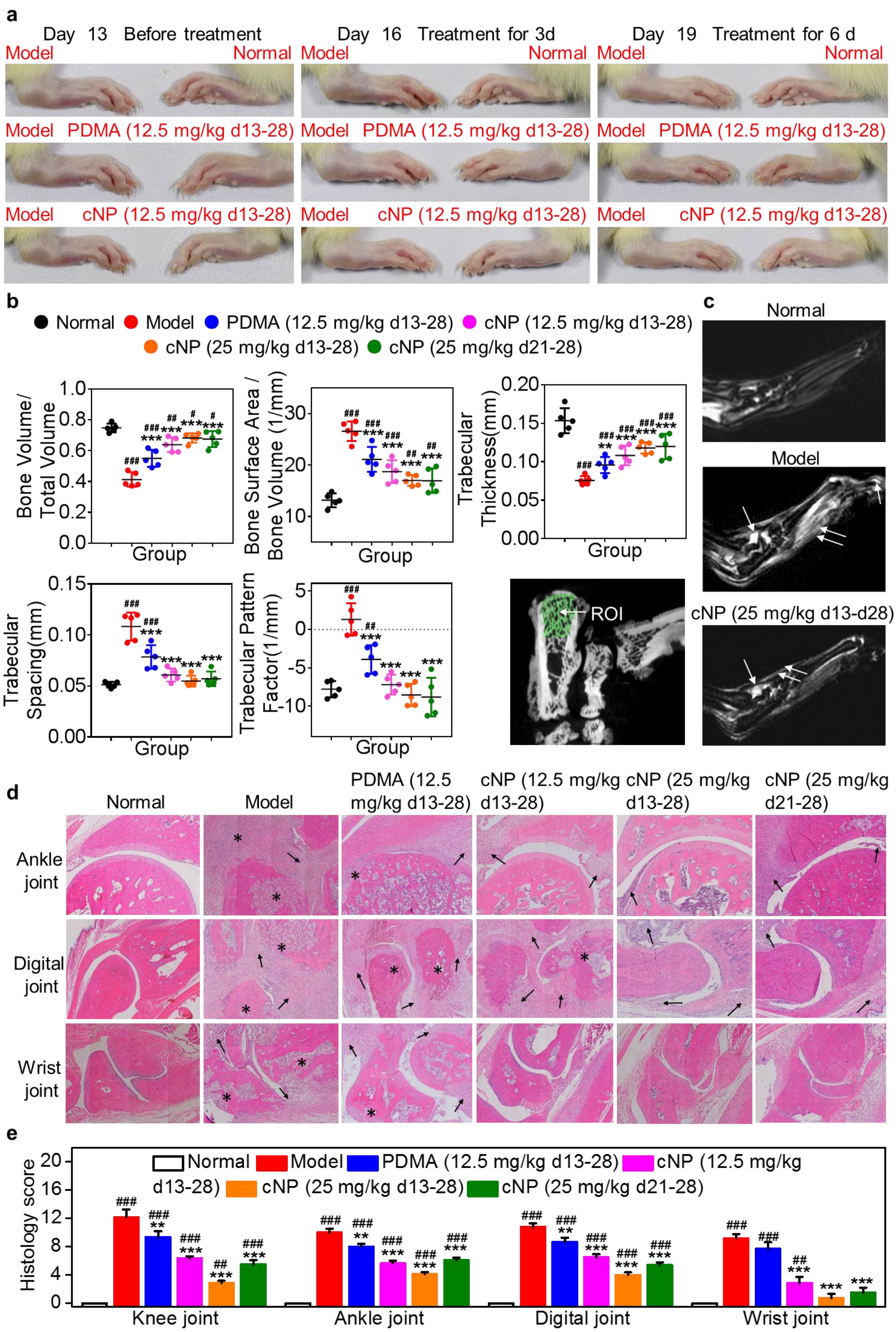

Supplementary Figure 8. Morphology and iconography study in both early and established therapeutic treatment of rats. a, Side views of hindpaw swelling of rats at different time points. **b,** Analyses of bone histomorphometric parameters of the ankle joint from rats after 15-day treatment obtained from the Micro-CT data. The bone trabecular of calcaneus was chosen as ROI. **c,** Representative T2-weighted MRI images

of the ankle joints of CIA rats at day 24. The effusion in the joint (single arrow) and tissue swelling (double arrow) was visible in the RA model, while treatment of cNP efficiently limited these pathological changes. **d,** Representative H&E staining of the ankle joints, the digital joints and the wrist joints of rats at day 29 (×200). Arrow indicates inflammatory cell infiltration, and * indicates bone destruction. **f,** Average histology scores of knee joints, ankle joints, digital joints and wrist joints of rats in different groups. In **b** and **e**, statistical significance was calculated by one-way ANOVA with the LSD post-test, *** P<0.001 versus the model group, # 0.01<P<0.05, ## 0.001<P<0.01, ### P<0.001 versus the normal group. Data are presented as the mean ±s.e.m.

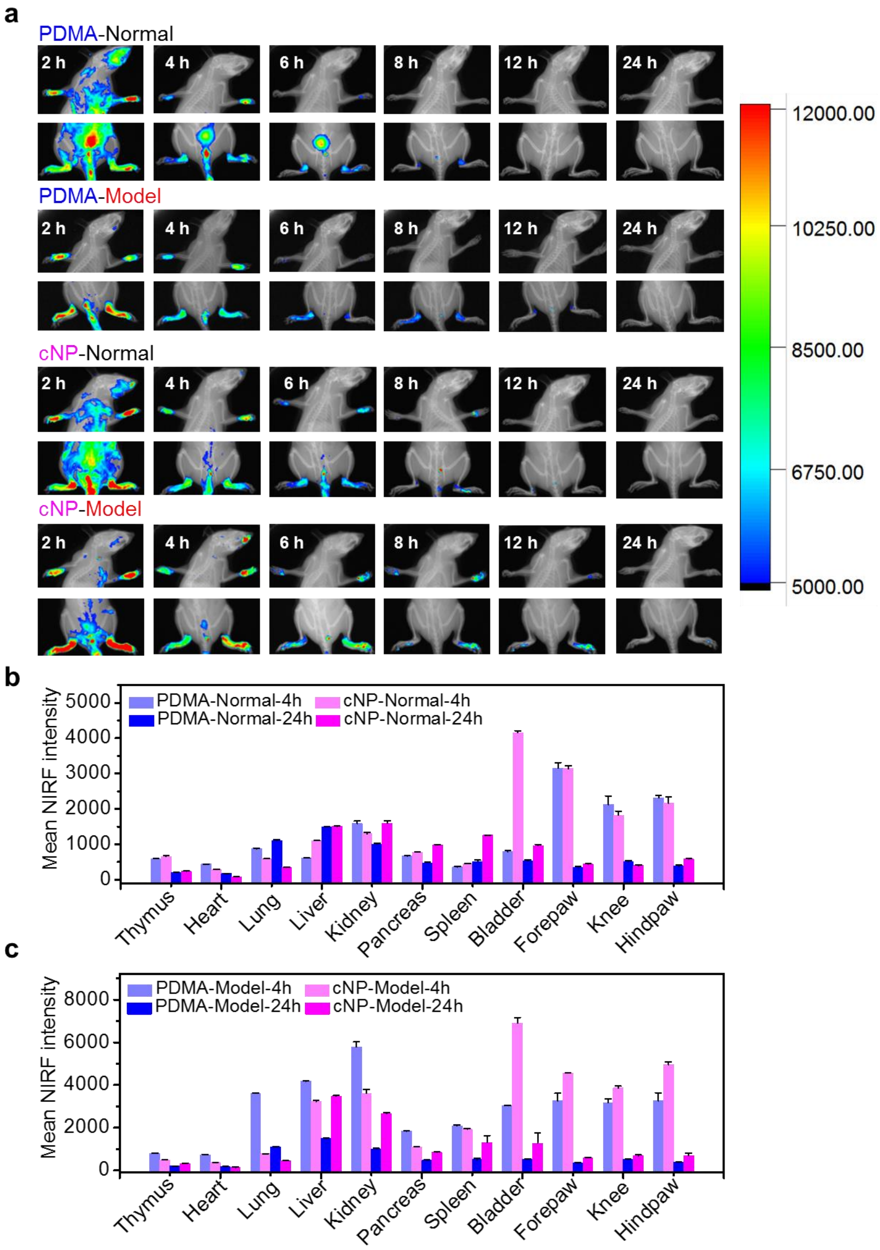

Supplementary Figure 9. Biodistribution of cationic materials in normal rats and CIA rats (score 2) via i.v. injection. a. In vivo NIRF images at 2, 4, 6, 8, 12 and 24 h demonstrated that the cationic materials could accumulate in joints, and persisted for at least 12 h. Note: the NIR fluorescence from major organs may be shielded because of the fur. **b** and **c**. Quantification of mean NIRF intensity of cationic polymers in ex vivo biodistribution study of normal rats (**b**) and model rats (**c**).

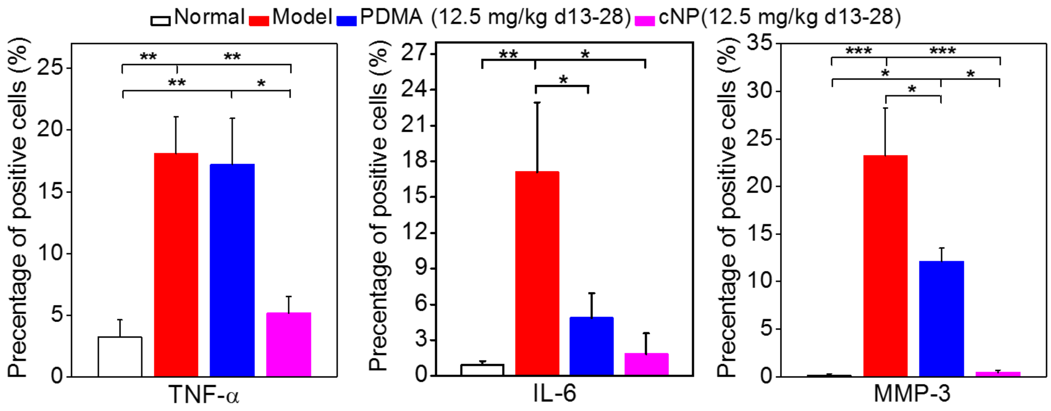

Supplementary Figure 10. Quantification of TNF-a, IL-6 and MMP-3 immunoreactive cells in synovial tissue of normal, model, PDMA, and cNP groups. The positive cells (brown staining) expressing TNF-α, IL-6 and MMP-3 in synovial tissue of different groups were analyzed by Nuance 3.0.2 and Inform 2.1.1 software. Statistical significance was calculated by one-way ANOVA with the LSD post-test, * 0.01<P<0.05, ** 0.001<P<0.01, *** P<0.001. Data are presented as the mean ± s.e.m.

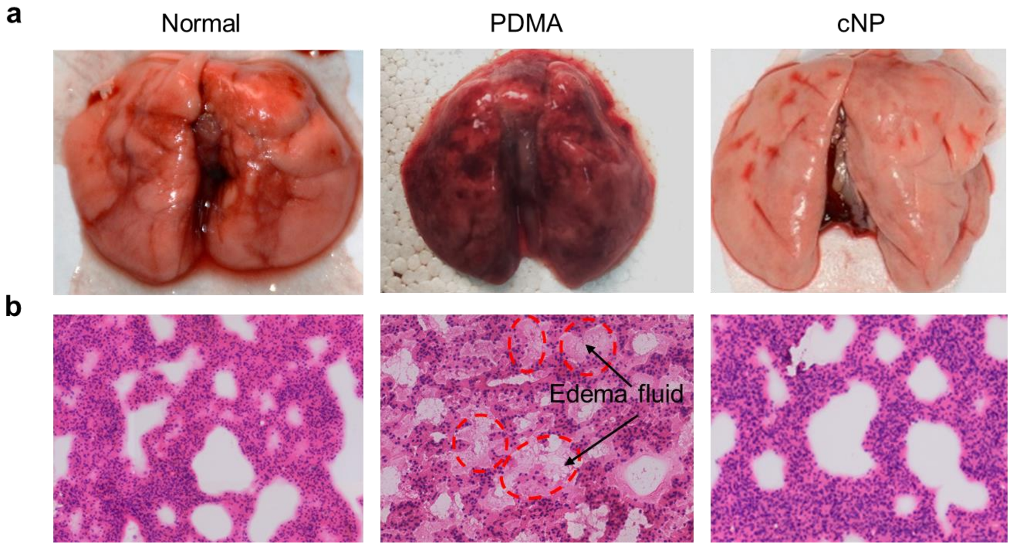

Supplementary Figure 11. In vivo acute toxicity study of cationic polymers in rats. **a,** Picture of lungs of rats after i.v. injection of 25 mg/kg cationic polymer for 24 h (PDMA group died in 10 min). The healthy rat died within 10 min after injection with 25 mg/kg PDMA and its lung was bloodshot. **b,** H&E staining of lungs of rats after i.v. injection of 25 mg/kg cationic polymers for 24 h (while PDMA group died in 10 min) (×400). A large amount of edema fluid in pulmonary alveoli (red circle) could be observed in PDMA group.

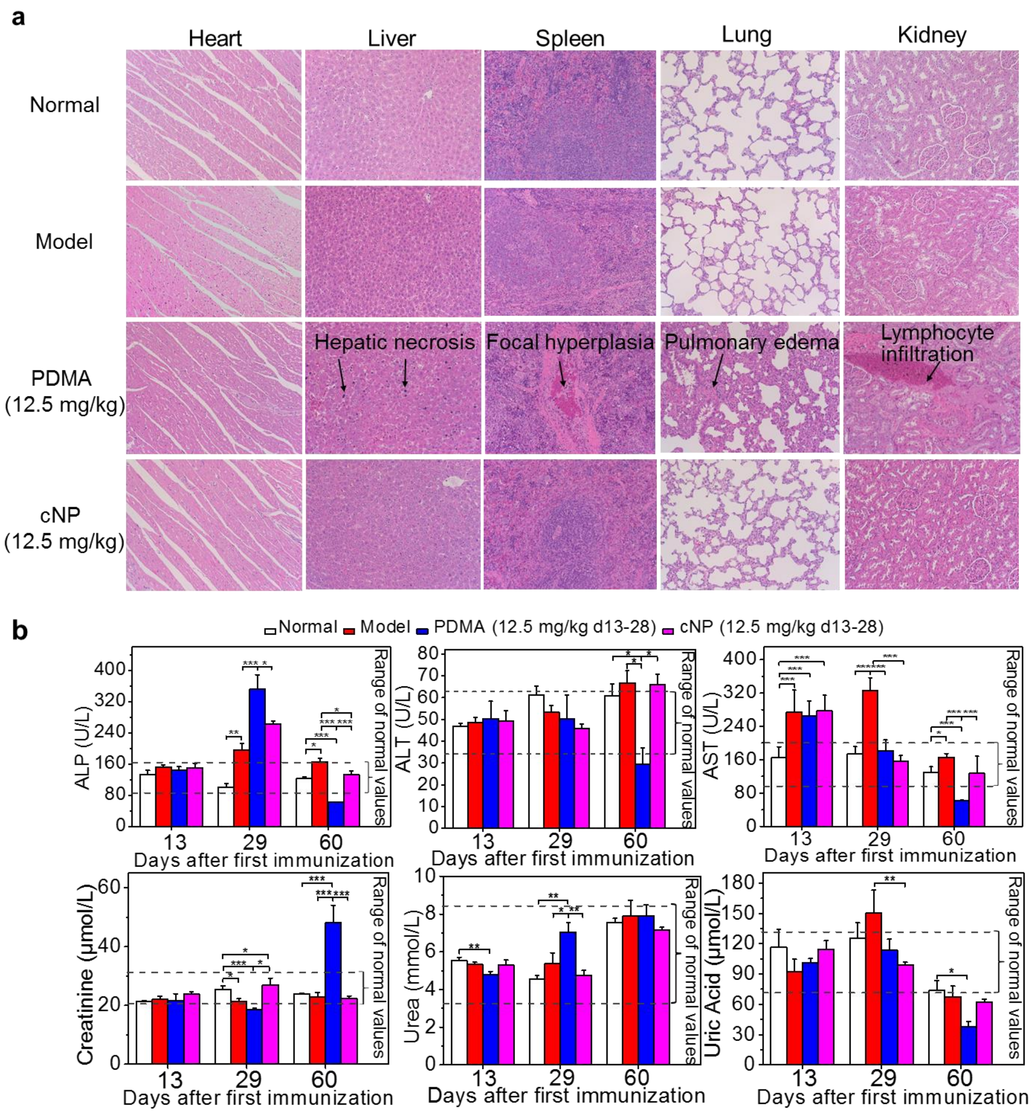

Supplementary Figure 12. In vivo long-term toxicity of cationic materials in rats. **a,** H&E staining of heart, liver, spleen, lung and kidney of rats. Lesions in liver, spleen, lung and kidney can be found in the PDMA group (×200). **b,** ALP, ALT, AST, creatinine, urea, and uric acid analysis of CIA rats before and after treating. Rat serum was collected at day 13 (before treatment), day 29 (treatment for 15 days), and day 60 (31 days after the last injection). The normal ranges of ALP, ALT and AST are 84-162, 34.6-64.0 and 96-200 U/L, respectively[1]. The normal ranges of creatinine, urea, and uric acid levels in rat serum are 20.17~30.50 μmol/L, 3.33~8.33 mmol/L and 71.4~133.28 μmol/L, respectively[1]. Statistical significance was calculated by one-way ANOVA with the LSD post-test, * 0.01<P<0.05, ** 0.001<P<0.01, *** P<0.001. Data are presented as the mean ± s.e.m.

Supplementary Table 1. Basic information and clinical characteristics of RA patients.

No.	1	2	3
Sex	Female	Female	Female
Age (years)	44	54	58
Weight (kg)	54	51	50
Smoking	No	No	No
Disease duration (years)	3	20	4
No. of arthroncus	4	2	6
CRP (mg/L)	5.4	30.3	79.8
ESR (mm/H)	72	120	23
ACPA	(-)	\	(-)
RF (IU/mL)	525	41.7	(-)

Supplementary References

1. Xu, S., Chen, X. *Methodology of Pharmacological Experiment*, **3**. People's Medical Publishing House Co., LTD: Beijing, (2001).
