# Supplementary material for: Cationic nanoparticle as an inhibitor of cell-free DNA-induced inflammation
Source: Nat Commun. 2018 Oct 16;9:4291. doi: 10.1038/s41467-018-06603-5 (PMC6191420; doi:10.1038/s41467-018-06603-5)
Supplement: Supplementary file 3 — Description of Additional Supplementary Files [file 41467_2018_6603_MOESM3_ESM.pdf]

Description of Additional Supplementary Files

File Name: Supplementary Movie 1
Description: The rotational test of rats at day 29 after first immunization.
